# GSTZ1 deficiency promotes hepatocellular carcinoma proliferation via activation of the KEAP1/NRF2 pathway

**DOI:** 10.1186/s13046-019-1459-6

**Published:** 2019-10-30

**Authors:** Jingjing Li, Qiujie Wang, Yi Yang, Chong Lei, Fan Yang, Li Liang, Chang Chen, Jie Xia, Kai Wang, Ni Tang

**Affiliations:** 1 Key Laboratory of Molecular Biology for Infectious Diseases (Ministry of Education), Institute for Viral Hepatitis, Department of Infectious Diseases, The Second Affiliated Hospital, Chongqing Medical University, Chongqing, China; 2 Department of Blood Transfusion, The Second Affiliated Hospital, Chongqing Medical University, Chongqing, China; 3 Institute of Life Sciences, Chongqing Medical University, Chongqing, China

**Keywords:** Glutathione S-transferase zeta 1, Hepatocellular carcinoma, Glutathione, Oxidative stress, KEAP1/NRF2 pathway

## Abstract

**Background:**

Glutathione S-transferase zeta 1 (GSTZ1) is the penultimate enzyme in phenylalanine/tyrosine catabolism. GSTZ1 is dysregulated in cancers; however, its role in tumorigenesis and progression of hepatocellular carcinoma (HCC) is largely unknown. We aimed to assess the role of GSTZ1 in HCC and to reveal the underlying mechanisms, which may contribute to finding a potential therapeutic strategy against HCC.

**Methods:**

We first analyzed GSTZ1 expression levels in paired human HCC and adjacent normal tissue specimens and the prognostic effect of GSTZ1 on HCC patients. Thereafter, we evaluated the role of GSTZ1 in aerobic glycolysis in HCC cells on the basis of the oxygen consumption rate (OCR) and extracellular acidification rate (ECAR). Furthermore, we assessed the effect of GSTZ1 on HCC proliferation, glutathione (GSH) concentration, levels of reactive oxygen species (ROS), and nuclear factor erythroid 2-related factor 2 (NRF2) signaling via gain- and loss- of GSTZ1 function in vitro. Moreover, we investigated the effect of GSTZ1 on diethylnitrosamine (DEN) and carbon tetrachloride (CCl_4_) induced hepatocarcinogenesis in a mouse model of HCC.

**Results:**

GSTZ1 was downregulated in HCC, thus indicating a poor prognosis. GSTZ1 deficiency significantly promoted hepatoma cell proliferation and aerobic glycolysis in HCC cells. Moreover, loss of GSTZ1 function depleted GSH, increased ROS levels, and enhanced lipid peroxidation, thus activating the NRF2-mediated antioxidant pathway. Furthermore, *Gstz1* knockout in mice promoted DEN/CCl_4_-induced hepatocarcinogenesis via activation of the NRF2 signaling pathway. Furthermore, the antioxidant agent N-acetylcysteine and NRF2 inhibitor brusatol effectively suppressed the growth of *Gstz1*-knockout HepG2 cells and HCC progression in *Gstz1*^−/−^ mice.

**Conclusions:**

GSTZ1 serves as a tumor suppressor in HCC. GSH depletion caused by GSTZ1 deficiency elevates oxidative stress, thus constitutively activating the NRF2 antioxidant response pathway and accelerating HCC progression. Targeting the NRF2 signaling pathway may be a promising therapeutic approach for this subset of HCC.

## Background

Hepatocellular carcinoma (HCC) is one of the most common malignant tumors worldwide [1]. However, despite advancements in surgery and traditional chemotherapy, its overall prognosis is still poor, especially in advanced HCC. Similar to all malignant tumors, HCC is a multi-step and multi-factorial disease. The Warburg effect, the first reported metabolic change in tumors, has provided novel insights into tumorigenesis, and metabolic reprogramming has been considered a new hallmark of cancer [2]. Hundreds of consistently deregulated metabolic genes have been identified in HCC in patients [3]. Restoration of these altered metabolic pathways may provide novel insights into cancer therapeutic approaches [4].

**Fig. 1:**
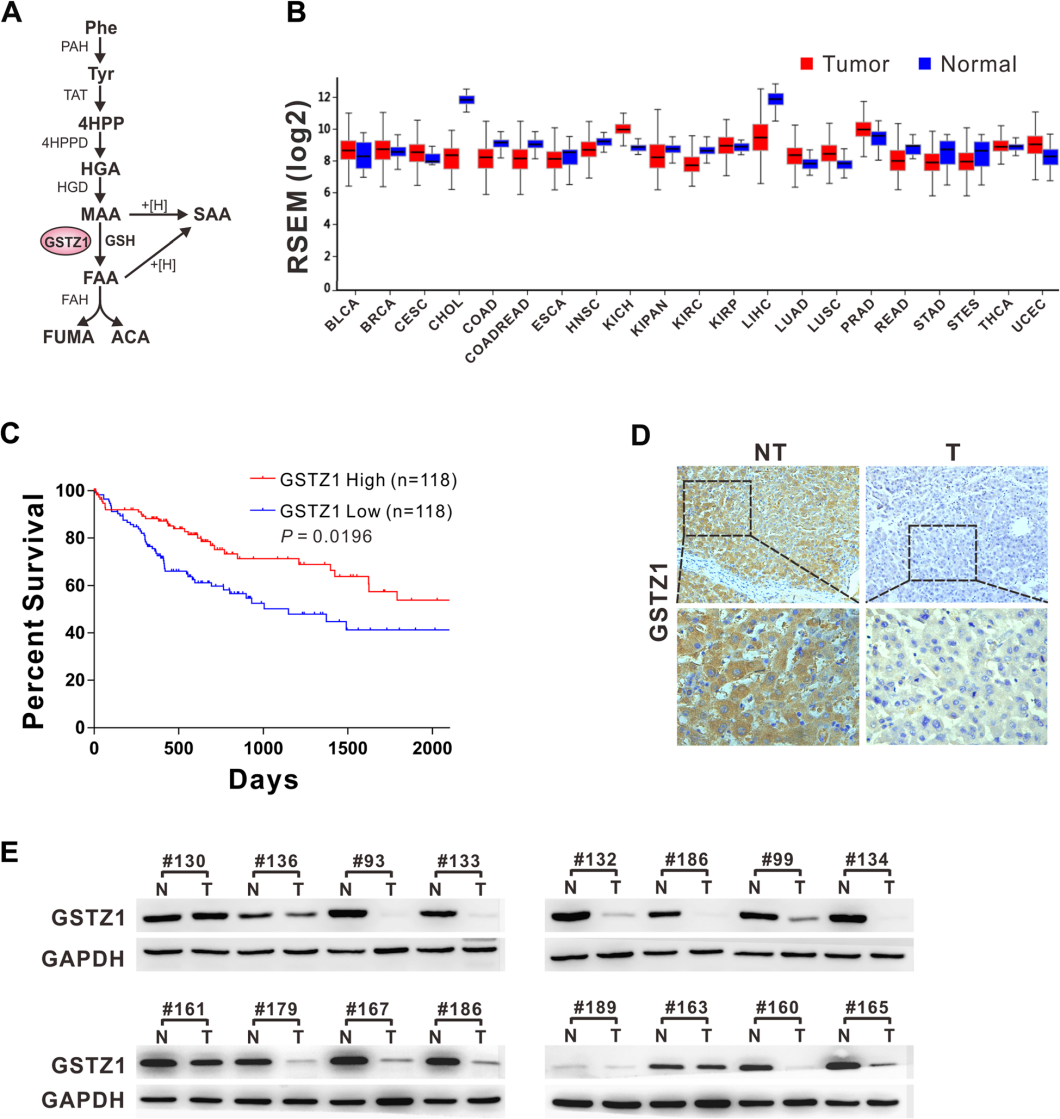
GSTZ1 is downregulated in human HCC tissues and correlates with poor survival of HCC patients. **a** Flow diagram of phenylalanine and tyrosine catabolism. **b** GSTZ1 differential plot. Levels of mRNA *Gstz1* expression in tumor tissues are shown with normal tissues for comparison. The colored bars represent tumor (red) and normal (blue) tissues. The data are derived from Firebrowse (http://firebrowse.org/). **c** Kaplan-Meier overall survival curve based on *GSTZ1* expression in TCGA LIHC datasets. Median values of overall survival were compared using the log-rank test. **d** Representative IHC images of GSTZ1 in HCC tissues and tumor-adjacent normal tissues. Magnifications: 200× and 400×. **e** GSTZ1 expression in 16 cases of HCC and paired non-tumor tissues. For Western blotting, 50 μg protein was loaded per well. Values represent the mean ± standard deviation (SD) (*n* = 3, performed in triplicate). PAH, 4-phenylalanine monooxygenase; TAT, tyrosine aminotransferase; 4HPP, 4-hydroxyphenylpyruvate; 4HPPD, 4-hydroxyphenylpyruvate dioxygenase; HGA, homogentisate; HGD, 1,2-homogentisate dioxygenase; MAA, maleylacetoacetate; FAA, fumarylacetoacetate; FAH, fumarylacetoacetate hydrolase; FUMA, fumarate; ACA, acetoacetate; SAA, succinylacetoacetate; NT, non-tumor; T, tumor; RSEM, RNA-seq by expectation maximization; IHC, immunohistochemistry

Glutathione *S*-transferase zeta 1 (GSTZ1), also known as maleylacetoacetate isomerase (EC 5.2.1.2), which catalyzes the glutathione-dependent isomerization of maleylacetoacetate (MAA) to fumarylacetoacetate (FAA) [5], is the penultimate enzyme in phenylalanine and tyrosine (Phe/Tyr) catabolism (Fig. 1a). Glutathione-S-transferase (GST) catalyzes the conjugation of glutathione to electrophilic endogenous or exogenous compounds, thus playing a critical role in cellular detoxification [6]. As a member of the GST family, GSTZ1 also catalyzes the oxygenation of dichloroacetic acid to glyoxylic acid and may be involved in xenobiotic α-haloacid metabolism [7].

*Gstz1-*knockout mice have reportedly displayed rapid weight loss, renal and hepatic damage, necrosis, and lethality, when stressed with a high phenylalanine diet [8]. GSTZ1 was recently reported to be downregulated in HCC and upregulated in breast cancer [9], indicating that GSTZ1 dysregulation may be involved in tumorigenesis in humans. However, the underlying mechanism remains largely unknown.

As a consequence of metabolic and signaling aberrations, cancer cells generally have elevated levels of reactive oxygen species (ROS), which are balanced by an increased antioxidant capacity [10]. The nuclear factor erythroid 2-related factor 2 (NRF2) is a master regulator of detoxification and the antioxidant response. NRF2 induces tumorigenesis, metastasis, and chemotherapeutic resistance [11]. Under normal conditions, NRF2 levels are retained at low levels by the E3 ubiquitin ligase Kelch-like ECH-associated protein 1 (KEAP1), which ubiquitinates NRF2 in the cytoplasm and targets it for degradation. Oxidative stress alters the conformation of KEAP1 via oxidation of cysteine residues, thus dissociating it from NRF2. NRF2 then translocates into the nucleus to activate its target genes, such as NAD(P)H: quinone oxidoreductase 1 (*NQO1*), heme oxygenase (*HO-1*)*,* glutamate-cysteine ligase catalytic subunit (*GCLC*), and glutamate-cysteine ligase modifier subunit (*GCLM*), through binding with the antioxidant response element (ARE) in their promoter regions [12, 13].

In the present study, we aimed to investigate the role of GSTZ1 in HCC and elucidate the underlying mechanisms. We evaluated the role of GSTZ1 in aerobic glycolysis in HCC cells on the basis of the oxygen consumption rate (OCR) and extracellular acidification rate (ECAR) and assessed its effect on HCC proliferation, GSH concentration, ROS levels, and NRF2 signaling via gain and loss of GSTZ1 function in vitro. Furthermore, we investigated the effect of GSTZ1 on diethylnitrosamine (DEN) and CCl_4_-induced hepatocarcinogenesis in a mouse model of HCC. The present study may yield a potential therapeutic strategy against HCC.

## Methods

### Patient samples for gene and protein expression analysis

Human HCC and adjacent non-tumor samples (surgical samples collected with informed consent from patients) were obtained from the first and second affiliated hospital of Chongqing Medical University. The samples were frozen immediately after surgery and stored in liquid nitrogen. The study was reviewed and approved by the Research Ethics Committee of Chongqing Medical University.

### Cell cultures

Human hepatoma cell lines HepG2, SK-Hep1, and PLC/PRF/5 were obtained from the American Type Culture Collection (Manassas, VA, USA). Other hepatoma cell lines including Huh7 and normal human hepatocytes MiHA were obtained from the Cell Bank of the Chinese Academy of Sciences (Shanghai, China). Apart from HepG2, cells were cultured in Dulbecco’s modified Eagle’s medium (Hyclone, Logan, UT, USA) supplemented with 10% fetal bovine serum (FBS; Gibco, Rockville, MD, USA), 100 IU penicillin, and 100 mg/ml streptomycin. HepG2 cells were cultured in Minimum Essential medium (MEM, Hyclone). These cell lines have been recently authenticated through short tandem repeat profiling (Beijing Microread Gene Technology Co., Beijing, China). All cells were incubated in a humidified atmosphere at 37 °C containing 5% CO_2_.

### TCGA data analysis

*Gstz1* mRNA expression data were obtained from The Cancer Genome Atlas (TCGA) dataset and analyzed using Firebrowse (http://firebrowse.org/). To compare expression levels, we used RNA-Seq by Expectation Maximization to determine the transcript abundance of genes. Kaplan-Meier survival analysis was performed via patient stratification based on *Gstz1* mRNA expression as high (top 33%) or low (bottom 33%).

### Construction of HCC cell lines overexpressing GSTZ1

The full-length cDNA of *GSTZ1* (NM_145870.2) was amplified from plasmid pOTB7-GSTZ1 (FL09522; GeneCopoeia, Guangzhou, Guangdong, China) and inserted into the *BamH* I and *Hind* III sites of the shuttle vector pAdTrack-TO4 (from Dr. T-C He, University of Chicago, Chicago, IL, USA). Adenoviral recombinant pAd-GSTZ1 was generated using the AdEasy system [14]. HCC cell lines endogenously expressing GSTZ1 at low levels, including Huh7, SK-Hep1, and MHCC-97H, were infected with AdGSTZ1 to establish GSTZ1-overexpressing cell lines. An analogous adenovirus expressing only GFP (AdGFP) was used as the control.

### CRISPR-Cas9 mediated GSTZ1-knockout in HepG2 cells

The E-CRISP online tool (http://www.e-crisp.org/E-CRISP/designcrispr.html) was used to design the *GSTZ1*-targeting sequences. The 20-bp *GSTZ1* targeting sequence selected herein, 5′- GCCCAGAACGCCATCACTTG-3′, immediately preceding a 5′-TGG-3′ protospacer adjacent motif, was derived from exon 6 of *GSTZ1*. Thereafter, the synthesized oligos were annealed and cloned into lentiCRISPR v2 plasmid in accordance with the method of the Zhang lab (http://genome-engineering.org). The CRISPR/Cas9 plasmids lentiCRISPR v2, pMD2.G, and psPAX2 were kindly provided by Prof. Ding Xue from the School of Life Sciences, Tsinghua University (Beijing, China). LentiCRISPR v2, envelop plasmid pMD2.G, and packaging plasmid psPAX2 were co-transfected into HEK293T cells to generate lentiviruses, using lipofectamine 2000, in accordance with manufacturer’s protocol. HepG2 cells were infected with the lentivirus, followed by puromycin selection to establish a stable *GSTZ1*-knockout (*GSTZ1*-KO) cell line. Thereafter, 96-well plates were used to select single-cell clones via the double dilution method. Clonal cell genomic DNA was extracted and cloned into the pMD19-T TA cloning vector and sequenced. The *GSTZ1* knockout efficiency was confirmed through Western blotting.

### Quantitative reverse transcription polymerase chain reaction (qRT-PCR) analysis

Total RNA was extracted using TRIzol (Invitrogen, Carlsbad, CA, USA) and reverse-transcribed to cDNA using PrimeScript RT reagent kit (Takara, Shiga, Japan) in accordance with the manufacturer’s instructions. Among all primers used herein, only the qPCR primer for TXN was the exon-spanning type. To minimize genomic DNA contamination, all RNA samples were digested with RNase-free DNase (Promega, Madison, WI, USA) and re-purified using mini columns prior to reverse transcription and qPCR. Furthermore, we used a non-RT negative control to monitor the quality of the experiment. Real-time qPCR was performed to quantify mRNA levels, using the iTaq Universal SYBR Green Supermix in accordance with the manufacturer’s instructions. Each 10-μL PCR reaction system comprised the following: 5 μL SYBR Green Supermix, 0.5 μL forward primer (10 μmol/L), 0.5 μL reverse primer (10 μmol/L), 2 μL cDNA, and 2 μL nuclease-free water. PCR was carried out using Bio-Rad CFX Connect Real-time PCR Detection System (Bio-Rad, Hercules, CA, USA) with the following reaction conditions: 95 °C for 30 s, followed by 35 cycles at 95 °C for 10 s, 62 °C for 30 s, and 72 °C for 30 s. Data were acquired during the extension step. The objective CT values were normalized to that of β-actin and the relative expression levels of genes were determined using the ΔΔCT method. The primer sequences and the accession numbers of individual genes are provided in Additional file 1: Table S1.

### Western blotting

Total proteins were obtained from cells of tissues, using Cell lysis buffer for Western blotting and IP (Beyotime, Shanghai, China), containing 1% of phenylmethanesulfonyl fluoride (PMSF) and cytoplasmic and nuclear proteins were extracted using the Nuclear and Cytoplasmic Protein Extraction Kit (Beyotime) in accordance with the manufacturer’s instructions. The protein concentration of the homogenates was measured using the BCA protein assay Kit (Dingguo, Beijing, China) in accordance with the manufacturer’s instructions. Protein lysates were boiled in SDS loading buffer for 5 min. The same amount of proteins (30 to 50 μg) were separated via sodium dodecyl sulfate-polyacrylamide gel electrophoresis (8–10% resolving gel) at 120 V for 60–90 min. Thereafter, the proteins were electro-transferred onto a polyvinylidene difluoride membrane (Millipore, Boston, MA, USA). After blocking with 5% non-fat milk dissolved in TBST (10 mM Tris, 150 mM NaCl, and 0.1% Tween-20; pH 7.6), the membranes were probed with the following primary antibodies overnight at 4 °C at a relative dilution rate in BSA: anti-GSTZ1 (1:1000; GTX106109, GeneTex, California, USA), anti-NQO1 (1:1000; ab34173, Abcam, Cambridge, UK), anti-GAPDH (1:5000; ab8245, Abcam), anti-β-actin (1:5000; ab6276, Abcam), anti-Lamin B1 (1:5000; ab133741, Abcam), anti-β-tubulin (1:1000; ab108342, Abcam), anti-NRF2 (1:1000; ab62352, Abcam), and anti-4HNE (1:1000; ab46545, Abcam) antibodies. Thereafter, membranes were washed thrice in TBST, followed by incubation with the secondary antibody (1:5000 to 1:10000) conjugated with horseradish peroxidase (HRP) for 1 h at room temperature. Membranes were then washed thrice with TBST before enhanced chemiluminescence (ECL) exposure.

### Immunohistochemistry (IHC)

The paraffin-embedded sections of tissues were incubated at 55 °C for 2–4 h, de-paraffinized in xylene for 20 min, and rehydrated successively in 100, 95, 85, and 75% ethanol and distilled water. After antigen retrieval with citrate buffer, the samples were incubated with 3% hydrogen peroxide for 10 min. Thereafter, samples were rinsed with PBS and blocked with goat serum for 1 h at room temperature. Residual serum was eliminated, and the samples were incubated with the following primary antibodies overnight at 4 °C at a relative dilution rate in PBS: anti-GSTZ1 (1:200), anti-NQO1 (1:200), and anti-Ki67 (1:100; ab15580, Abcam) antibodies. After rinsing with PBS, samples were incubated with reagent A of Elivision™ plus Polyer HRP (Mouse/Rabbit) IHC Kit (Maixin, Fuzhou, China) for 20 min, followed by incubation with reagent B, secondary antibody conjugated with HRP for 30 min. Signals indicating HRP activity were visualized using DAB reagent. The nuclei were counterstained with hematoxylin for 2.5 min. After rinsing, the samples were dehydrated, treated with xylene for transparency, and observed using a microscope.

### Cell growth curve and clone formation assay

The proliferation capacity of HCC cells was assessed using a cell growth curve and by assessing the clone formation capacity. Cells were seeded into a 96-well plate (2000–3000 cells/well), with three replicate wells per group. The cells were incubated in the ESCO Cell culture CO_2_ Incubator (Esco Technologies, Inc., Horsham, PA, USA) for 120 h and enumerated automatically every 24 h. The growth curves were plotted using GraphPad Prism 7 software (GraphPad Software Inc., San Diego, CA, USA).

Cells were seeded in a 6-well plate (2000 cells/well), with three replicate wells per group, and cultured at 37 °C and 5% CO_2_. After 7–10 d, the cells were fixed with 4% paraformaldehyde for 30 min and incubated with Crystal Violet. After rinsing thrice with PBS, the clones were photographed and enumerated.

### EdU assay

The proliferation capacity of HCC cells was assessed using the Cell-Light™ EdU DNA cell proliferation kit (RiboBio, Guangzhou, Guangdong, China), in accordance with the manufacturer’s instructions. Cells were seeded on coverslips in a 6-well plate. Before detection, cells were incubated with Reagent A at a dilution of 1:1000 in culture medium for 2 h. After fixing with 4% paraformaldehyde, cells were incubated with fluorescent dye (Apollo 643) for 30 min. DAPI was used to counterstain the nuclei. Thereafter, the coverslips were mounted on glass slides with Anti-fade Mounting Medium (Beyotime). Images were captured using a Leica confocal microscope (TSC SP8) with a 40× objective lens. The percentage of EdU-positive cells was determined for comparison.

### Quantification of oxygen consumption and the extracellular acidification rate

The OCR and ECAR of Huh7 cells and HepG2 cells were determined using the Seahorse Bioscience Extracellular Flux Analyzer (XFe24; Seahorse Bioscience). Cells were plated at 50,000 cells/well with 250 μL culture medium in a 24-well cell culture plate (Seahorse Bioscience), and incubated at 37 °C, 5% CO_2_ overnight. Thereafter, adherent cells were washed and replaced with 500 μL XF Base Medium pH 7.4 (Seahorse Bioscience) supplemented with 1 mM sodium pyruvate, 2 mM glutamine, and 10 mM glucose. Cells were incubated at 37 °C in a non-CO_2_ incubator for 1 h. The hydrated XF24 sensor cartridge was loaded oligomycin (1 μM final concentration) and protonophore trifluoromethoxy carbonyl cyanide phenylhydrazone (FCCP, 1 μM final concentration). Thereafter, basal OCR and ECAR were measured in accordance with the manufacturer’s instructions.

### ARE promotor activity

The promotor activity of the antioxidative response element (ARE) was detected using the Dual-Luciferase Reporter System (Promega, Madison, WI, USA). The ARE reporter plasmid pGL3-ARE (see Additional file 2: Figure S1 for the plasmid map) was kindly provided by Prof. Yiguo Zhang from Chongqing University. Plasmids pGL3-ARE (3 μg) and pRL-TK (0.3 μg) were co-transfected into cells cultured in a 6-cm dish. After 24 h, the cells were re-seeded into 24-well plates, three replicate wells for each group. Thirty-six hours later, the cells were lysed with 1 × Passive Lysis Buffer (100 μL/well) for 15 min on shaker at room temperature. The lysates were centrifuged, and the supernatants were harvested. Thereafter, Firefly and Renilla luciferase activities were analyzed in accordance with the manufacturer’s instructions. Relative luciferase activities, reflecting the promotor activities, were determined by normalizing firefly luciferase activity to that of the corresponding Renilla luciferase.

### Gstz1^−/−^ mouse in vivo assay

Heterozygous 129-*Gstz1*^tm1Jmfc^/Cnbc mice (EM: 04481) were purchased from European Mouse Mutant Archive via Nanjing Biomedical Research Institute of Nanjing University and were crossed to breed wild-type (WT) and *Gstz1*^*−/−*^ mice. Before hepatic GSH levels were determined via mass spectrometry, the mice were fed with 2% phenylalanine for 1 week. For subsequent analysis, mice were divided into groups as follows: WT (control), *Gstz1*^*−/−*^, *Gstz1*^*−/−*^ + NAC, and *Gstz1*^*−/−*^ + Brusatol. Each group included ten mice, 5 female and 5 male. Mice of all groups were intraperitoneally administered DEN at 75 mg/kg at 2 weeks of age [15]. At the third week, the mice were fed with 0.25% Phe until euthanasia and intraperitoneally administered CCl_4_ at 2 mL/kg twice a week for 12 weeks. At 20 weeks of age, another dose of DEN (50 mg/kg) was intraperitoneally administered. In the *Gstz1*^*−/−*^ + NAC group, the *Gstz1*^*−/−*^ mice were administered 4 g/L NAC at 3 weeks until euthanasia. In the *Gstz1*^*−/−*^ + Brusatol group, the *Gstz1*^*−/−*^ mice were intraperitoneally administered brusatol at 2 mg/kg every 2 d, 2 weeks before euthanasia. Their body weight was determined every week. Blood was sampled from the tail vein and serum α-fetoprotein (AFP) levels were determined immediately before euthanasia. All mice were euthanized at 32 weeks of age, and their livers were dissected out. The liver weight and the number of hepatic tumors were determined. Protein and RNAs were extracted from hepatic tumors for Western blotting and qRT-PCR analysis, respectively. Hepatic tumors were fixed with 4% paraformaldehyde, embedded in paraffin, and sectioned for hematoxylin-eosin staining and immunohistochemistry. Hepatic tumor sections were further frozen for ROS detection. All animal procedures were performed in accordance with protocols approved by the Rules for Animal Experiments published by the Chinese Government and approved by the Research Ethics Committee of Chongqing Medical University (reference number: 2017010).

### Intracellular and in vivo ROS detection

Cells were seeded on coverslips in a 6-well plate. After 24 h, Cells were incubated with a fluorescence probe (CellROX Reagent Orange; Thermo, Wilmington, DE, USA) at a concentration of 5 μM in 1× PBS for 30 min at 37 °C (and protected against light). After washing thrice with 1× PBS, DAPI was used to counterstain the nuclei for 3 min. Thereafter, cells were washed, and the coverslips were mounted on glass slides with Anti-fade Mounting Medium. Images were acquired using Leica Confocal Microscope 1 (TSC SP8) with a 60× oil immersion objective lens.

The frozen sections were prepared from hepatic tumors of 129 SvJ mice immediately after euthanasia. The frozen sections were gently washed with 1× PBS and incubated with a fluorescence probe (CellROX Reagent Orange; Thermo) at a concentration of 5 μM in 1× PBS for 30 min at 37 °C (and protected against light). After three gentle washes with 1× PBS, DAPI was used to counterstain nuclei for 3 min. Thereafter, the sections were washed and mounted with Anti-fade Mounting Medium. Images were acquired using Leica Confocal Microscope (TSC SP8) with a 20× objective lens.

### GSSG/GSH assay

Cells were transfected with pEIGW Grx1-roGFP2 plasmid (Addgene #64990). The intracellular ratio of oxidized GSH disulfide (GSSG) to reduced GSH (GSSG/GSH) was detected as previously described [16]. Briefly, Grx1-roGFP2-expressing cells were excited with lasers at 405 and 488 nm, followed by emission at 505–550 nm detected using a confocal microscope (Leica TSC SP8). Raw data were exported to ImageJ software (National Institutes of Health, Bethesda, MD, USA). GSSG/GSH ratios were determined through pixel-by-pixel division of the image acquired at 405 nm with that acquired at 488 nm. Mouse liver tissues (approximately 100 mg) were used for GSH measurement. Liver tissues were extracted with cold acetonitrile/water (4:1, v/v) using a homogenizer, and GSH concentrations were measured via ultra-high performance liquid chromatography coupled with a triple-quadrupole mass spectrometer (UHPLC-QqQ-MS).

### Statistical analysis

Statistical analysis and data plotting were performed using GraphPad Prism 7 (GraphPad Software Inc., San Diego, CA, USA). Data were presented as mean ± standard deviation (SD) values. Unless mentioned otherwise, the Student’s *t*-test was used for between-group comparisons, and the one-way ANOVA analysis followed by the Tukey test for post hoc analysis was used for multiple-group comparisons. Statistical significance was defined as *p* < 0.05.

## Results

### GSTZ1 downregulation in HCC predicts a poor patient prognosis

To evaluate intratumor GSTZ1 deregulation, we compared its expression between tumor tissue and tumor-adjacent normal tissue samples with mRNA-Seq data from TCGA database using Firebrowse (Fig. 1b). As shown in the differential plot, GSTZ1 was markedly downregulated in liver hepatocellular carcinoma (LIHC). We further evaluated the prognostic impact of GSTZ1 on patients with primary HCC in TCGA-LIHC. Kaplan-Meier’s survival curve analysis revealed that patients with low GSTZ1 expression levels had a significantly shorter median overall survival than those with high expression (median survival, 1149 versus 2116 days, *p* = 0.0196; Fig. 1c).

Further, we examined GSTZ1 expression in 16 paired clinical HCC and normal liver tissue samples via IHC and Western blotting, which revealed that GSTZ1 was significantly downregulated in HCC rather than tumor-adjacent normal tissue (Fig. 1d-e). These data indicate that GSTZ1 downregulation in HCC may contribute to disease progression and predict a poor prognosis.

### GSTZ1 suppresses the proliferation of hepatoma cells

**Fig. 2:**
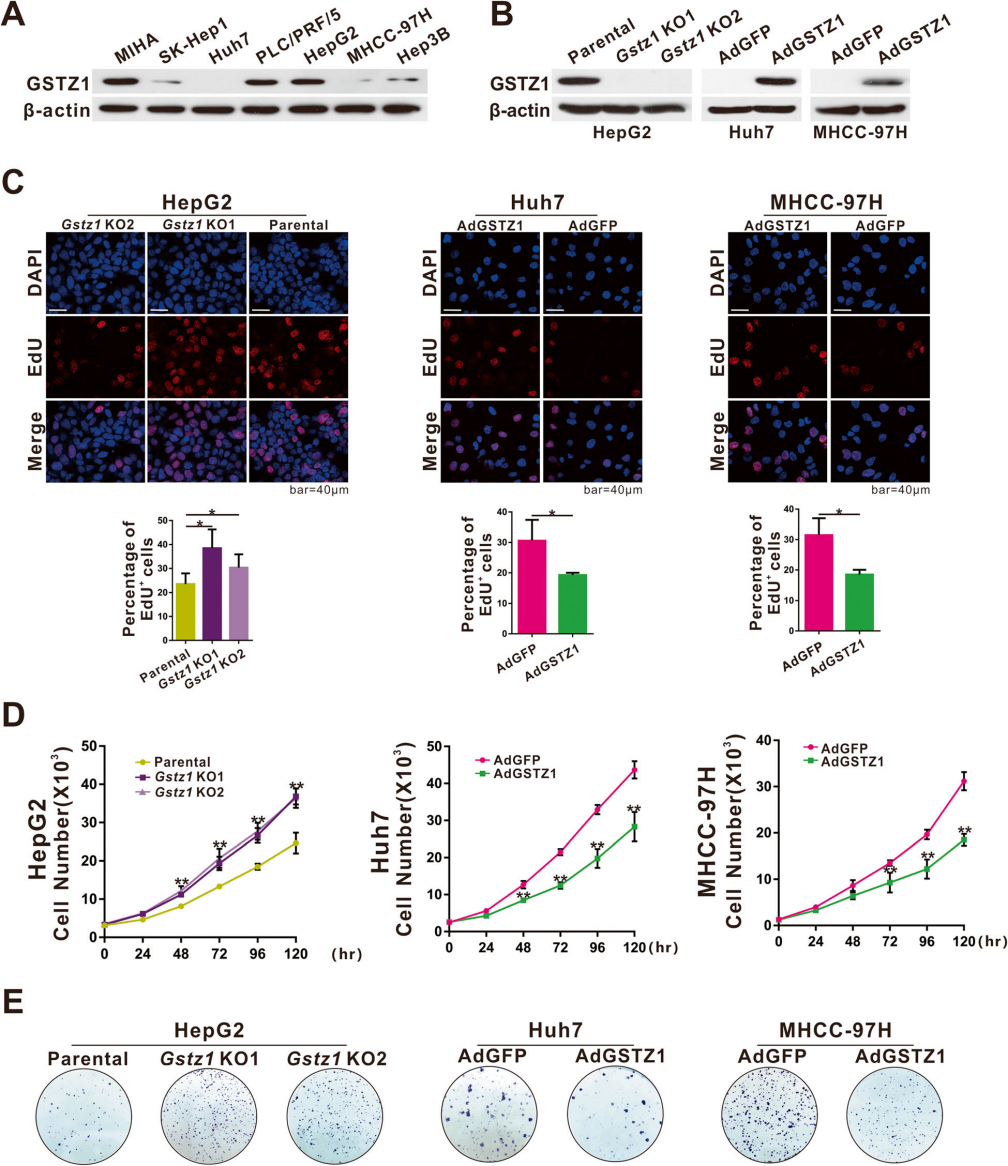
GSTZ1 inhibits cell proliferation in HCC cell lines. **a** Western blotting showing endogenous GSTZ1 protein expression in HCC cell lines. **b** GSTZ1 overexpression in Huh7 and MHCC-97H cells, and knockout (KO) of *GSTZ1* in HepG2 cells were confirmed through immunoblotting. The overexpression model was established by infecting hepatoma cells with adenoviruses expressing GSTZ1 (AdGSTZ1), and adenoviruses expressing GFP (AdGFP) was used as a control. The knockout model was established with the CRISPR-Cas9 system. **c-e** The proliferation potential of GSTZ1-overexpressing Huh7, MHCC-97H cells, and *GSTZ1*-KO HepG2 cells. **c** Representative images and enumeration of EdU-positive cells. **d** Cell growth curve. **e** Representative images and determination of the colony formation capacity. For Western blotting, 50 μg protein was loaded per well. Values represent the mean ± SD (*n* = 3, performed in triplicate), *p < 0.05, **p < 0.01, Student’s *t*-test (two groups) or one-way ANOVA followed by Tukey tests (three groups). HCC, hepatocellular carcinoma

To investigate the role of GSTZ1 in HCC, we first determined endogenous GSTZ1 protein levels in some hepatoma cell lines. Western blotting revealed that GSTZ1 was substantially downregulated in most hepatoma cells, consistent with our previous study [14]. Among the HCC cell lines, Huh7, MHCC-97H, and SK-Hep1 cells expressed GSTZ1 at markedly lower levels than HepG2 and PLC/PRF/5 cells, wherein endogenous GSTZ1 expression was robust (Fig. 2a). To evaluate the effect of GSTZ1 on cell proliferation, we infected Huh7 and MHCC-97H cells with adenoviruses containing cDNA constructs encoding GSTZ1 (AdGSTZ1), and the empty adenovirus control (AdGFP). Furthermore, the stable HepG2^*Gstz1−/−*^ cell line was established, in which the *Gstz1* gene was knocked out using the CRISPR/cas9 system. Overexpression and knockout efficiencies were confirmed through Western blotting (Fig. 2b). GSTZ1 overexpression significantly suppressed the proliferation of both Huh7 and MHCC-97H cells, as revealed through EdU incorporation, cell growth curves, and colony formation assays, whereas *GSTZ1* knockout promoted HepG2 cell proliferation (Fig. 2c-e).

### GSTZ1 suppresses the Warburg effect and ROS production in hepatoma cells

**Fig. 3:**
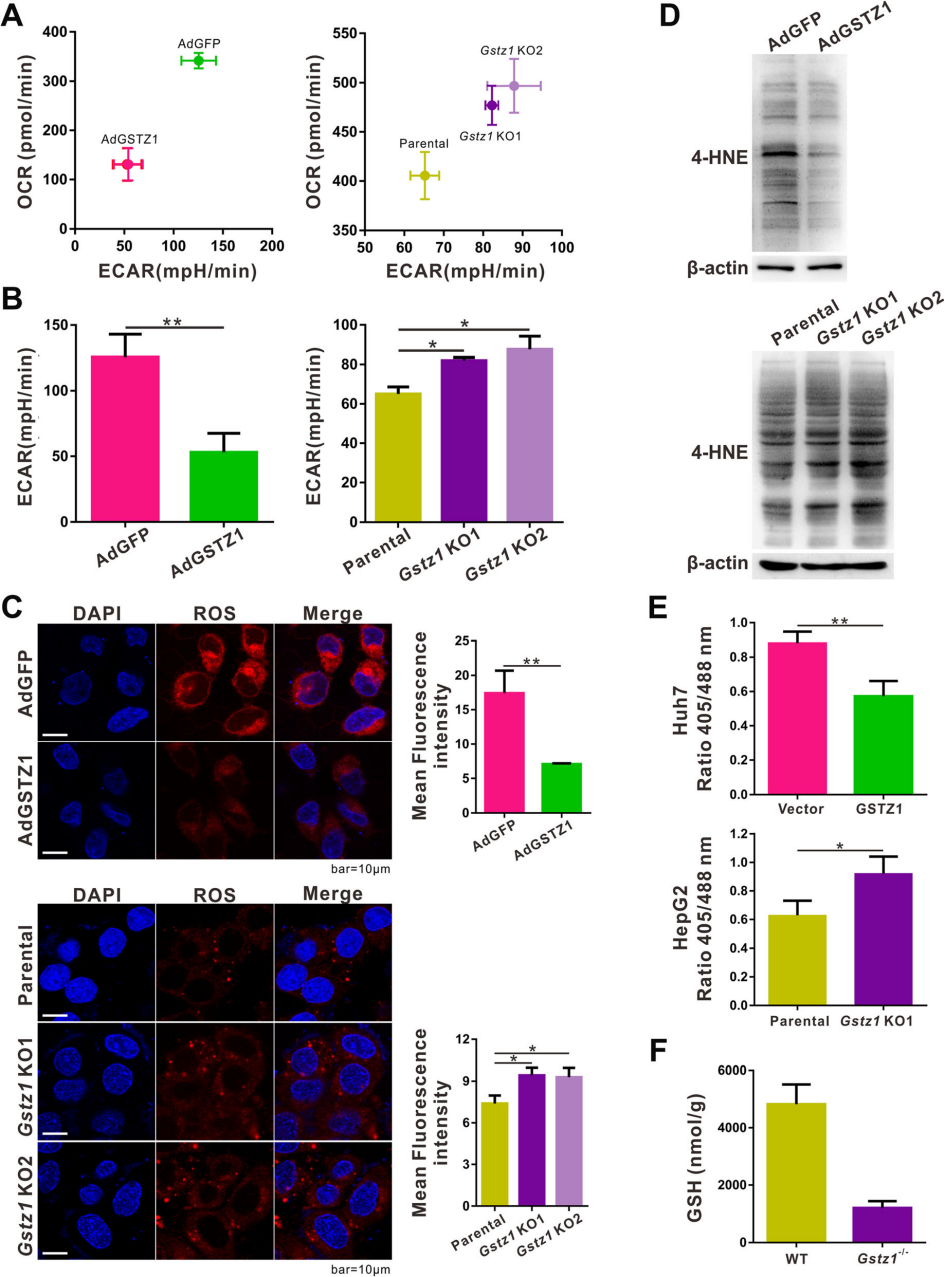
GSTZ1 suppresses the Warburg effect and ROS accumulation in HCC cell lines. **a**-**b** The ECAR and OCR in GSTZ1-overexpressing Huh7 cells (left) and *GSTZ1*-KO HepG2 cells (right) were determined using the Seahorse XF Cell Energy Phenotype test. GSTZ1-overexpressing Huh7 cells were less glycolytic than AdGFP control cells. In contrast, *GSTZ1*-KO HepG2 cells were more glycolytic than parental cells. **a** OCR versus ECAR. **b** ECAR values. **c** Representative fluorescence images (left) and quantification (right) of ROS levels in GSTZ1-overexpressing SK-Hep1 (top) and *GSTZ1*-KO HepG2 cells (bottom) stained with the CellROX Orange fluorescence probe. Immunofluorescence intensity was quantified using ImageJ. Values represent mean ± SD values. **d** Western blotting revealed the levels of 4-HNE modification in proteins in GSTZ1-overexpressing Huh7 cells (top) and *GSTZ1*-KO HepG2 cells (bottom). **e** GSSG/GSH ratio for GSTZ1-overexpressing Huh7 cells (top) and G*STZ*1-KO HepG2 cells (bottom) determined with Grx1-roGFP2. **f** Liver GSH levels in wildtype and *Gstz1*^*−/−*^ mice, detected via mass spectrometry. For Western blotting, 50 μg protein was loaded per well. Values represent mean ± SD values (*n* = 3, performed in triplicate), *p < 0.05, **p < 0.01, Student’s *t*-test (two groups) or one-way ANOVA followed by Tukey tests (three groups). ECAR, extracellular acidification rate; OCR, oxygen consumption rate; HCC, hepatocellular carcinoma; ROS; reactive oxygen species; GSH, glutathione; GSSG: GSH disulfide

Since GSTZ1 is a metabolic enzyme and most cancer cells perform aerobic glycolysis, we investigated whether GSTZ1 affects the metabolic state of hepatoma cells, using a Seahorse Bioscience XFe24 analyzer. Baseline OCR and ECAR were plotted as an energy map. Huh7 cells infected with AdGSTZ1 were less energetically active than those infected with the AdGFP control, whereas *Gstz1*-KO cells had a more active metabolic phenotype than parental HepG2 cells (Fig. 3a). Moreover, overexpression of GSTZ1 (*p* < 0.01, Fig. 3b left) significantly decreased the glycolytic rate of hepatoma cells, whereas *Gstz1*-KO (*p* < 0.05, Fig. 3b right) had the opposite effect.

ROS are byproducts of oxidative metabolism that are primarily produced in the mitochondria [17]. We examined ROS production in hepatoma cells with different GSTZ1 expression levels. ROS production was significantly reduced in *GSTZ1*-overexpressing SK-Hep1 cells (*p* < 0.01, Fig. 3c, top) and significantly increased in *GSTZ1*-KO HepG2 cells (*p* < 0.05, Fig. 3c bottom), compared to parental cells. A major electrophilic byproduct of lipid peroxidation, 4-Hydroxy-2-nonenal (4-HNE), is a biomarker of oxidative stress [18]. Concurrently, 4-HNE-modified protein levels were reduced in *GSTZ1*-overexpressing Huh7 cells and elevated in *GSTZ1*-KO HepG2 cells (Fig. 3d).

GSH, the most abundant intracellular antioxidant, is oxidized to GSSG during oxidative stress [19]. To determine the effect of GSTZ1 on GSH levels in HCC cells, we determined the intracellular GSSG/GSH ratios with a redox-sensitive biosensor, Grx1-roGFP2. The GSSG/GSH ratio was significantly decreased in *GSTZ1*-overexpressing Huh7 cells (*p* < 0.01, Fig. 3e top) and significantly increased in *GSTZ1*-KO HepG2 cells (*p* < 0.05, Fig. 3e bottom). Similar results were observed with an animal model, and GSH levels in the liver of 129SvJ background *Gstz1*^*−/−*^ mice were significantly lower than those in wildtype mice, as determined through mass spectrometry (*p* < 0.01, Fig. 3f).

### Gstz1 deficiency activates the KEAP1/NRF2 pathway

**Fig. 4:**
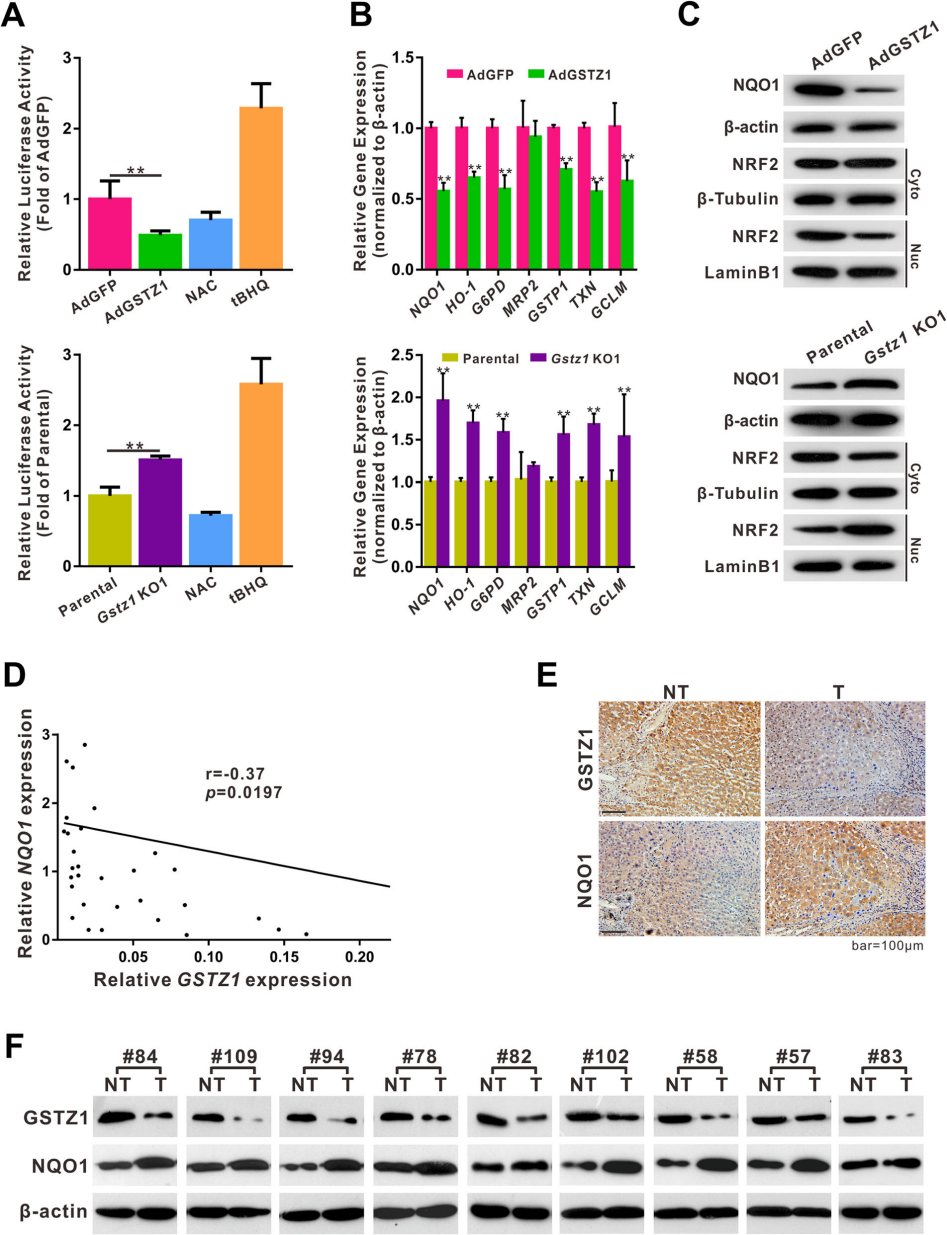
GSTZ1 negatively regulates the KEAP1/NRF2 pathway in human HCC and in liver tissue of mice. **a**–**c** ARE promoter activities in GSTZ1-overexpressing Huh7 cells (left) and *GSTZ1*-KO HepG2 cells (right). **a** ARE promoter activities. The NRF2 activator tBHQ (100 μM for 6 h) and the antioxidant NAC (4 mM for 24 h) were used as positive and negative controls, respectively. **b** Relative mRNA expression of NRF2 target genes in GSTZ1-OE and *GSTZ1*-KO HCC cells, determined via qRT-PCR. **c** Western blotting to assess expression levels of NQO1, the NRF2 target, in total cell extracts, and of NRF2 in cytoplasmic and nuclear cell extracts, indicating the effect of GSTZ1 on NRF2 intracellular localization and transcription. β-actin, β-tubulin, and Lamin B1 served as reference proteins for total, cytoplasmic, and nuclear extracts, respectively. **d**-**f** Association between GSTZ1 and NQO1 expression in HCC tissues. **d** Linear regression analysis showing the negative correlation between *GSTZ1* and *NQO1* mRNA expression in 40 cases of HCC (*p* = 0.0197; *r* = − 0.37, Pearson correlation coefficient). **e** Representative immunohistochemistry images for GSTZ1 and corresponding NQO1 in HCC tissues and tumor-adjacent normal tissues. **f** Western blotting for GSTZ1 and NQO1 in 9 pairs of HCC and tumor-adjacent normal tissues. For Western blotting, 50 μg protein was loaded per well. Values represent the mean ± SD (*n* = 3, performed in triplicate), *p < 0.05, **p < 0.01, Student’s *t*-test. ARE, antioxidant response element; HCC, hepatocellular carcinoma; qRT-PCR, quantitative reverse transcription polymerase chain reaction

*Gstz1*-knockout mice reportedly exhibit increased oxidative stress and activation of NRF2-mediated antioxidant response pathways [20]. To verify this observation, we evaluated the effect of GSTZ1 on the NRF2 antioxidant pathway. The effect of GSTZ1 on the expression of ARE-dependent genes was analyzed using an ARE-regulated luciferase reporter plasmid (pGL3-ARE) in hepatoma cells. Moreover, the subcellular localization of NRF2 and mRNA expression of NRF2 target genes were evaluated. GSTZ1 overexpression decreased NRF2 nuclear translocation, ARE luciferase activity, and the expression of NRF2 target genes, including *NQO1*, *HO-1*, *G6PD*, *GSTP1*, *GCLM*, and *TXN* (Fig. 4a-c). In contrast, *GSTZ1* knockout activated the NRF2 antioxidant pathway (Fig. 4a-c). To confirm this observation, we conducted RT-qPCR analysis using 40 tumor samples from HCC patients to detect *GSTZ1* and *NQO1* mRNA expression, and we observed a negative correlation between *GSTZ1* and *NQO1* expression (*r* = − 0.37, *p* = 0.0197, Fig. 4d). Western blotting and IHC revealed increased levels of NQO1 and decreased levels of GSTZ1 in most tumors, compared to tumor-adjacent normal tissues (Fig. 4e and f). Together, these data indicate that GSTZ1 deficiency activates the KEAP1/NRF2 pathway and promotes NQO1 expression in HCC.

**Fig. 5:**
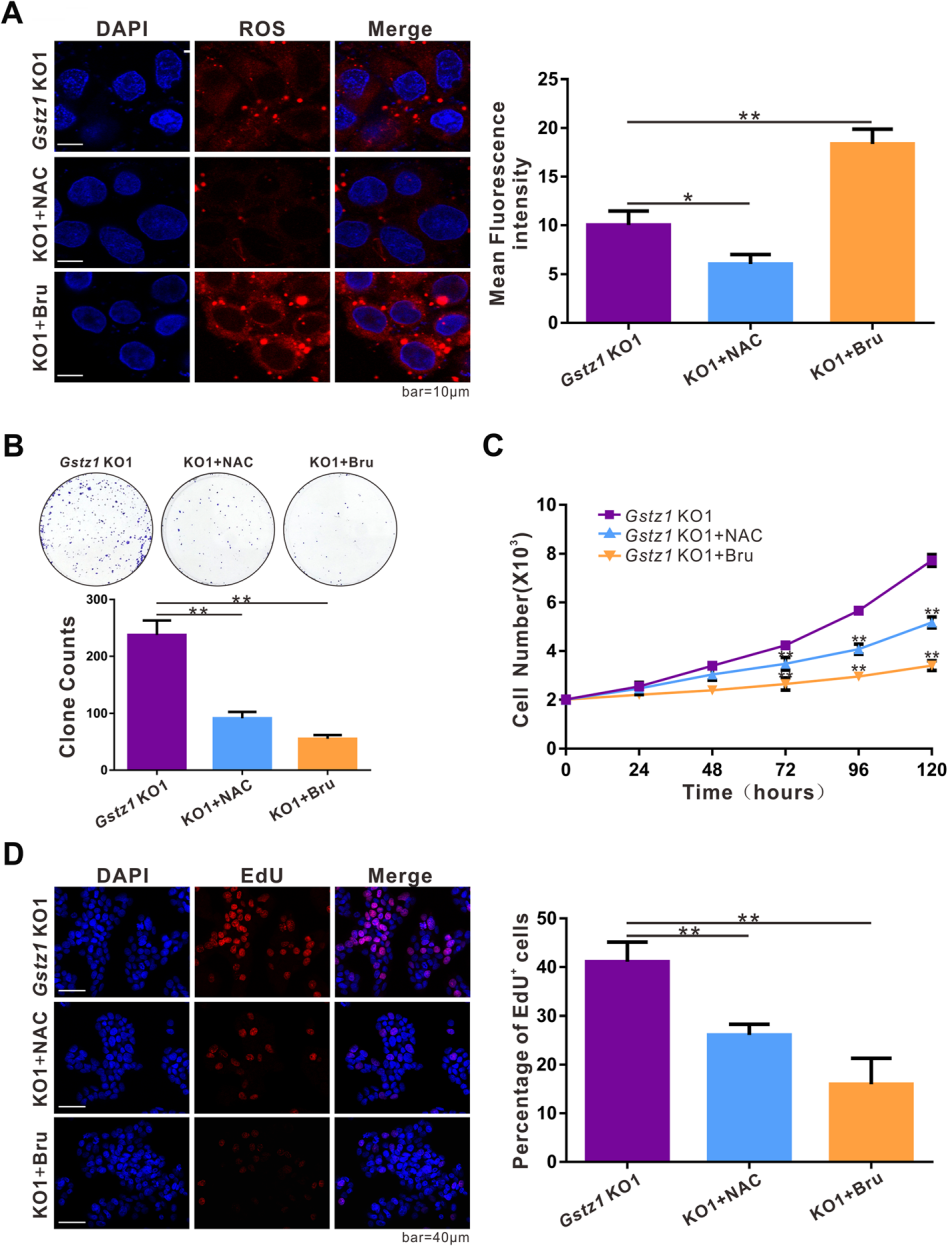
GSTZ1 deficiency promotes HCC cell growth via activation of the KEAP1/NRF2 pathway. **a** ROS levels in *Gstz1*-KO1 cells treated with antioxidant NAC for 24 h or NRF2 inhibitor brusatol for 12 h. **b-d** Effect of NAC or Bru on the proliferation potential of *Gstz1*-KO1 cells. **b** Representative images and colony formation potential. **c** Cell growth curve. **d** Representative images (left) and enumeration (right) of EdU positive cells. The final concentrations of NAC and brusatol were 4 mM and 40 nM, respectively. Values represent means ± SD (*n* = 3, performed in triplicate), **p < 0.01, one-way ANOVA test. HCC, hepatocellular carcinoma; ROS, reactive oxygen species

Since oxidative stress and constitutive NRF2 activation are implicated in cancer cell growth and survival, modulation of the NRF2 antioxidant pathway has emerged as a promising approach for cancer therapy [1]. Therefore, we investigated whether the antioxidant agent *N*-acetylcysteine (NAC) or the NRF2 inhibitor brusatol (Bru) could suppress proliferation in GSTZ1-deficient hepatoma cells. As expected, NAC significantly reduced ROS levels (*p* < 0.05, Fig. 5a) in *Gstz1*-KO HepG2 cells, whereas Bru significantly promoted ROS production (*p* < 0.01, Fig. 5a). Both NAC and Bru suppressed the activation of the NRF2-mediated signaling pathway, albeit through different mechanisms. Colony formation, cell growth curves, and EdU incorporation assays revealed that both NAC and Bru significantly inhibited the proliferation of *GSTZ1*-KO HepG2 cells in vitro (Fig. 5b–d).

### Gstz1 knockout promotes DEN/CCl_4_-induced hepatocarcinogenesis in mice

**Fig. 6:**
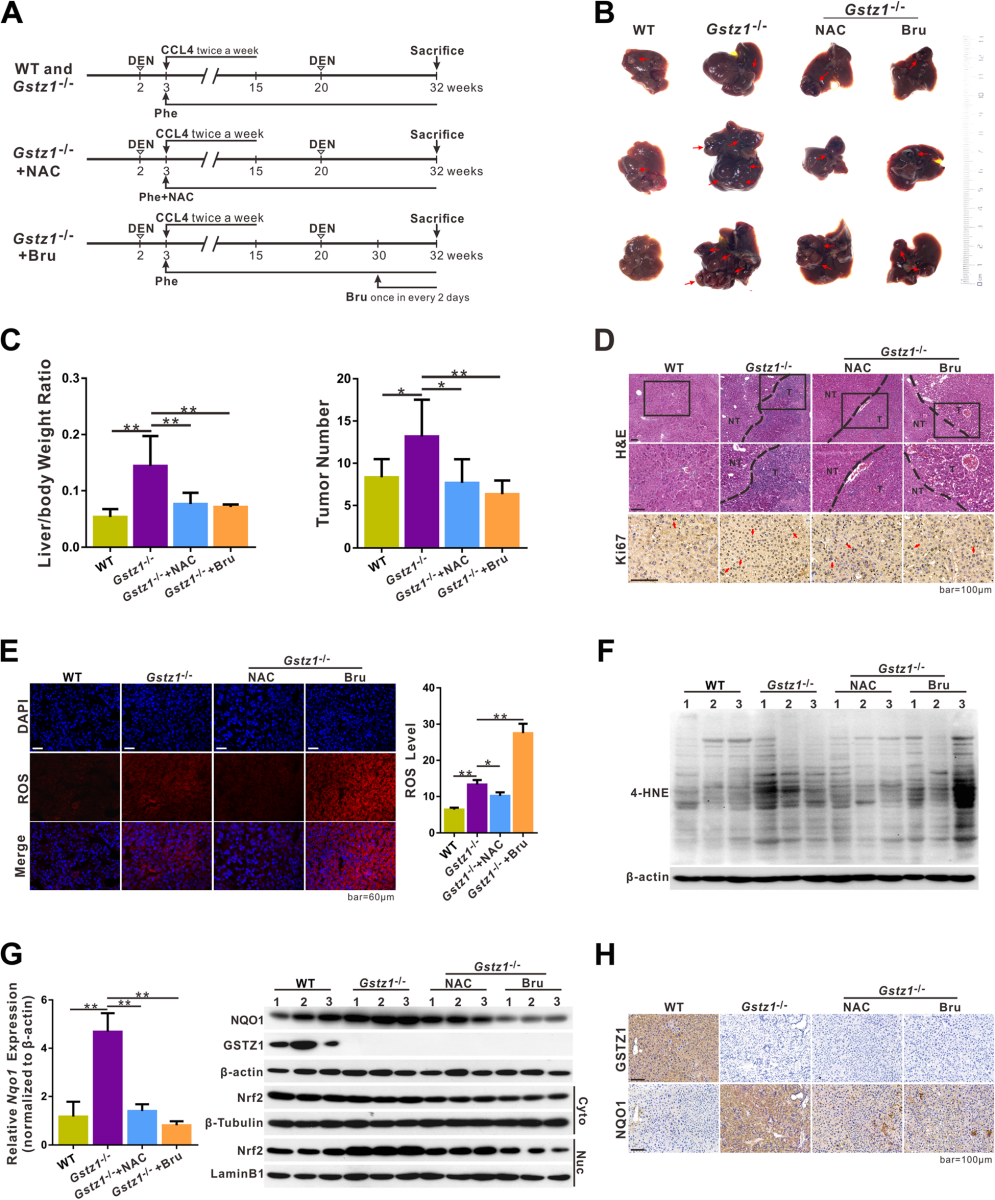
Knockout of *Gstz1* promotes DEN/CCl_4_-induced hepatocarcinogenesis in vivo via activation of the KEAP1/NRF2 pathway. **a** Schematic representations of the experimental design for WT and *Gstz1*^*−/−*^ mice. **b** Gross appearances of murine livers. Red arrows indicate tumors. **c** Liver/body weight ratio (left) and number of tumors (right) in each group. **d** Representative H&E staining and immunohistochemistry staining of Ki67 in liver tumors. **e-f** Oxidative stress levels in liver tumors. **e** Representative fluorescence staining of ROS with CellROX Orange probe in hepatic tumors of WT and *Gstz1*^/−^ mice (left). Intracellular ROS quantification (right). **f** 4-HNE modification is all proteins in tumor tissues, analyzed via Western blotting. **g** NRF2 transcriptional activities in liver tumors. Relative *Nqo1* mRNA expression, determined via qRT-PCR (Left). NQO1 expression in total cell extracts, and NRF2 expression in cytoplasmic and nuclear extracts, analyzed via Western blotting (Right). **h** Representative immunohistochemistry images of GSTZ1 and NQO1 in hepatic tumors. For Western blotting, 30 μg protein was loaded per well. Values represent the mean ± SD (*n* = 3, performed in triplicate), *p < 0.05, **p < 0.01, Student’s *t*-test (two groups) or one-way ANOVA followed by Tukey tests (four groups). H&E, hematoxylin and eosin; DEN, diethylnitrosamine; HCC, hepatocellular carcinoma; ROS, reactive oxygen species; 4-HNE, 4-hydroxy-2-nonenal; qRT-PCR, quantitative reverse transcription polymerase chain reaction

**Fig. 7:**
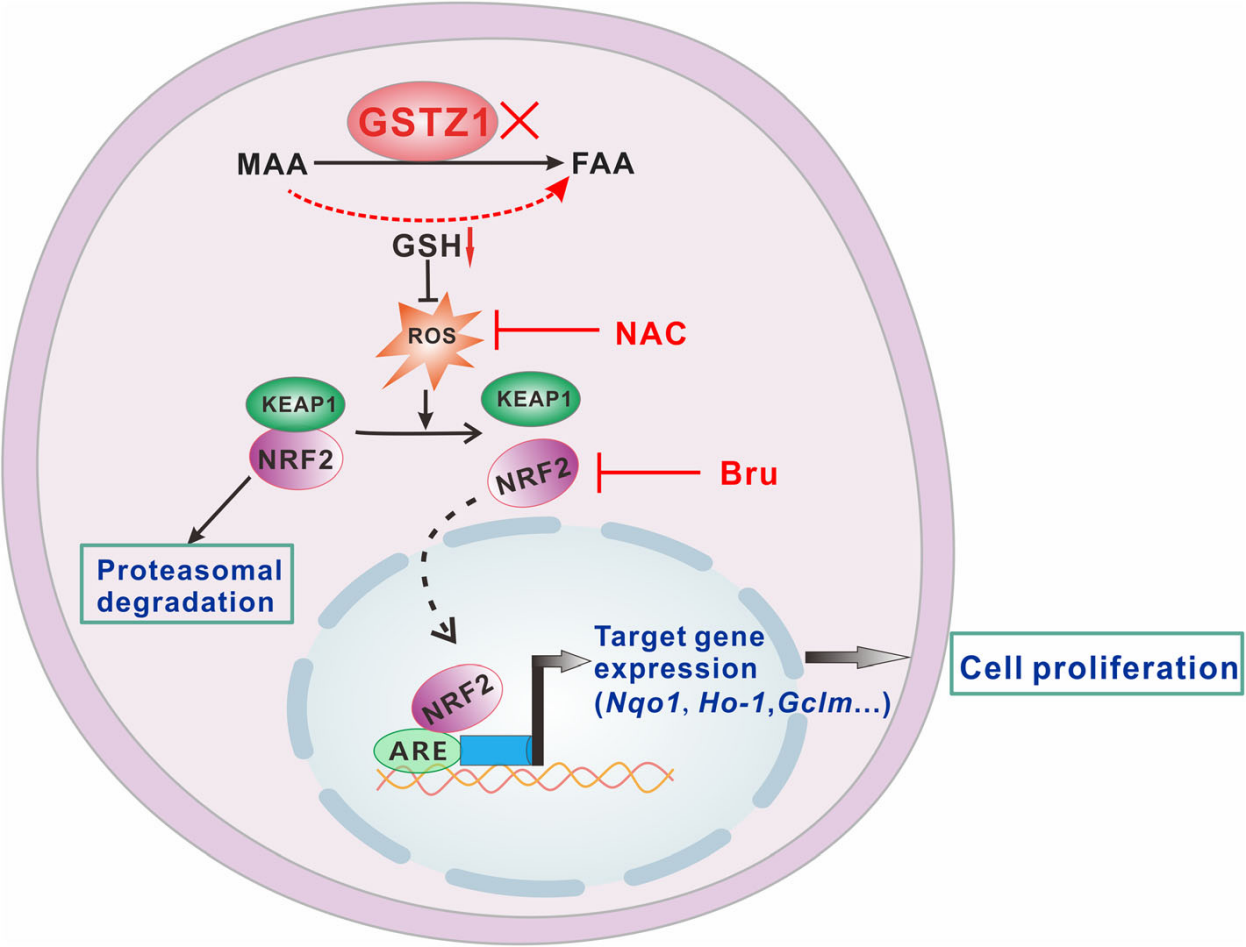
Proposed model for the activation of the KEAP1/NRF2 pathway by ROS. GSTZ1 deficiency activates the GSH-dependent non-enzymatic bypass to catalyze the conversion of MAA to FAA. The GSH-consuming reaction reduces the antioxidant capacity of cells, thus elevating ROS levels. Increased ROS levels impair the ability of KEAP1 to negatively regulate NRF2, leading to NRF2 nuclear translocation and subsequent transactivation, thus promoting HCC proliferation. ROS, reactive oxygen species; MAA, maleylacetoacetate; FAA, fumarylacetoacetate; GSH, glutathione. Bru, brusatol; NAC, N-acetylcysteine

To further investigate the role of GSTZ1 in hepatocarcinogenesis, wild-type (WT) and *Gstz1*^*−/−*^ mice were injected with DEN (75 mg/kg), followed by repeated administration of CCl_4_ (2 mL/kg twice a week for 12 weeks) and injected a second time with DEN (50 mg/kg) at 20 weeks of age (Figs. 6a and 7). All mice were administered 0.25% (w/v) Phe in the drinking water from 3 weeks postpartum to increase the burden on the Phe/Tyr catabolic pathway. Compared with WT mice, *Gstz1*^*−/−*^ mice exhibited increased liver tumor sizes and numbers of tumor nodules (Fig. 6b and c). Long-term administration of NAC water (4 g/L from 3 weeks postpartum until euthanasia) protected against tumorigenesis in *Gstz1*^*−/−*^ mice (Fig. 6b and c). Bru-treated *Gstz1*^*−/−*^ mice displayed smaller tumors and fewer tumor nodules than the untreated *Gstz1*^*−/−*^ mice. Histological analysis indicated that the nuclei of liver tumors in *Gstz1*^*−/−*^ mice exhibited strong immunoreactivity for Ki67 (Fig. 6d). However, either NAC or Bru significantly inhibited cell proliferation in *Gstz1*^*−/−*^ mice, which was highly consistent with the in vitro results.

Furthermore, *Gstz1*^*−/−*^ mice exhibited enhanced ROS accumulation and 4-HNE modification in their liver tumor tissues, compared with WT mice (Fig. 6e and f). Consistent with the in vitro results, NAC reduced ROS levels, whereas Bru promoted ROS production in hepatic tumors in *Gstz1*^*−/−*^ mice. NQO1 and nuclear NRF2 were markedly upregulated in hepatic tumors in Gstz1^−/−^ mice rather than in WT mice (Fig. 6g and h), which indicated sustained activation of the KEAP1/NRF2 pathway caused by GSTZ1 deficiency.

Together, these data indicate that *Gstz1* knockout increases the susceptibility to DEN/CCl_4_-induced hepatocarcinogenesis and progression in mice. GSTZ1 deficiency leads to GSH depletion, oxidative stress, and constitutive activation of the KEAP1/NRF2 pathway, thus accelerating the progression of hepatocarcinogenesis (Fig. 7).

## Discussion

The liver is a key metabolic site of aromatic amino acids such as phenylalanine and tyrosine [21]; their catabolic pathway is of intrinsic value and clinical interest. Deficiencies in the enzymes of this catabolic pathway cause various severe diseases leading to hepatotoxicity and nephrotoxicity, such as tyrosinemia [22]. Hereditary tyrosinemia is caused by deficiencies in enzymes involved in phenylalanine and tyrosine catabolism, such as fumarylacetoacetate hydrolase, tyrosine aminotransferase, or 4-hydroxyphenylpyruvic dioxygenase. Hereditary tyrosinemia type 1 (HT1) is characterized by progressive liver diseases with an increased risk of HCC [23, 24]. However, limited information is available regarding the role of GSTZ1, the penultimate enzyme of phenylalanine and tyrosine catabolism, in diseases thus far. Recently, six children with mild hypersuccinylacetonemia caused by sequence variants in *GSTZ1* were reported; however, no evidence of liver dysfunction was obtained [25].

The current study shows that *GSTZ1* is markedly downregulated in HCC, thus predicting a poor prognosis, concurrent with recent reports [3, 9]. In contrast, *GSTZ1* is reportedly upregulated in breast cancer [3, 9]. Several studies have highlighted dual roles for cancer-associated genes in different tumor types. Phosphoenolpyruvate carboxykinase, which converts oxaloacetate to phosphoenolpyruvate in the second step in gluconeogenesis, is reportedly upregulated in breast cancer, cervical carcinomas, and melanoma but downregulated in HCC [26]. Furthermore, GSTZ1 may play different roles in different tumor cell types, although limited information is available regarding its exact role in breast cancer [27, 28].

Our in vitro and in vivo gain- and loss-of-function studies revealed that GSTZ1 suppressed the Warburg effect and proliferation in hepatoma cells, indicating that GSTZ1 plays a tumor suppressor role in HCC. Our results suggest that GSTZ1 deficiency results in GSH depletion, followed by elevation of oxidative stress and sustained NRF2 activation, thus promoting proliferation in hepatoma cells. Intermediate metabolites of tyrosine catabolism accumulated in the urine of *Gstz1*^*−/−*^ 129Sv4 mice [8] and in the serum of *Gstz1*^*−/−*^ BALB/c mice [29]. These metabolites react with GSH to form stable adducts and decrease intracellular GSH levels [30]. However, GSH can mediate the isomerization of MAA to FAA independent of GSTZ1 under a GSTZ1-deficient condition [8]. Herein, GSH depletion was demonstrated in the *GSTZ1*-KO hepatoma cells and the liver of *Gstz1*^*−/−*^ mice, concurrent with our previous study on *Gstz1*^*−/−*^ mice [20]. However, baseline GSH levels were observed in mice deficient for fumarate hydratase [31], leading to fumarate accumulation; however, this enzyme does not depend on GSH. Therefore, our results can be explained on the basis of GSH consumption caused by the co-effect of metabolites adduction and non-enzymatic bypass reactions in tyrosine catabolism.

ROS, such as the hydroxyl radical (HO•) and superoxide anion (O_2_^−^), are generated by partial reduction of oxygen as byproducts of various cellular phenomena [32]. GSH is the most abundant antioxidant within cells and maintains cellular redox homeostasis. A reactive aldehyde, 4-HNE, is formed upon lipid peroxidation. Levels of global HNE-protein adducts are considered a biomarker for oxidative stress under various pathological conditions [33]. Herein, the generation of a constant level of oxidative stress, characterized by ROS and 4-HNE, in *Gstz1*^*−/−*^ mice may have resulted from sustained GSH consumption.

Under physiological conditions, ROS are maintained at low levels and serve as signaling molecules to activate cell proliferation and survival pathways [34]. Moderate ROS levels induce oxidative stress, resulting in DNA damage and genetic instability and contribute to tumor initiation and progression [10]. However, once ROS levels become excessively high, they can lead to cell death [35]. Cancer cells exhibit increased intrinsic ROS levels owing to uncontrolled proliferation and a high metabolic rate [11]. Therefore, antioxidant pathways need to be further induced in tumor cells to maintain favorably high ROS levels for survival and proliferation [35]. This study shows that oxidative stress was significantly reduced in GSTZ1-overexpressing hepatoma cells but elevated in *GSTZ1*-KO hepatoma cells. Furthermore, *Gstz1*^*−/−*^ mice exhibited enhanced ROS accumulation and 4-HNE modification in hepatic tumor tissues, compared with WT mice. These results indicate that GSTZ1 potentially plays a crucial role in regulating redox homeostasis in HCC.

NRF2 is a master regulator of the cellular antioxidant response. ROS activates NRF2 by promoting disulfide formation with KEAP1 [36]. Herein, GSTZ1 deficiency activated the NRF2 antioxidant pathway in vitro and in vivo. We speculate that the constant production of oxidative stress, resulting from GSTZ1 deficiency, may have activated the KEAP1/NRF2 pathway. NRF2 has been traditionally considered a tumor suppressor owing to its cytoprotective effects against damage from xenobiotics and oxidative stress. However, several studies have reported high constitutive activation of NRF2 in many tumors, such as carcinomas of the lungs, liver, gallbladder, ovary, breast, and stomach [37]. Concomitant to increased NRF2 activation, NRF2-target antioxidant enzymes such as GSTs and NQO1 are reportedly upregulated in cancer cells [38, 39]. NRF2 has recently been reported to promote cancer progression through metabolic reprogramming, apoptotic resistance, and metastasis promotion [11], further supporting our results that GSTZ1 deficiency promotes HCC proliferation and inhibition of the NRF2 pathway by NAC or Bru suppressed *Gstz1*^*−/−*^ hepatoma cell proliferation in vitro and in vivo. The present results show increased ROS levels and enhanced activation of the NRF2 antioxidant pathway. This controversy also exists with respect to FH-deficient HLRCC cells [40, 41]; however, the underlying mechanism remains unknown. We speculate that the antioxidant capacity of NRF2 may not be adequate to scavenge all augmented ROS, thus increasing ROS levels, which was beneficial to tumor progression under these circumstances.

As a member of the GST family, GSTZ1 expression may be stimulated by NRF2 interacting with antioxidant response elements (AREs) [42], through which GSTZ1 would be upregulated. However, several other transcription factors along with NRF2 also interact with AREs to regulate target genes, such as *NRF1*, *NRF3*, and *BACH1* [43]. Activation or suppression of target genes by NRF2 depends on dimerizing partners and AREs [43]. Moreover, NRF2 suppresses various targets [44]. No functional assays with AREs of *GSTZ1* have been reported thus far; however, Marhenke et al. reported that NRF2 does not affect *Gstz1* expression in the Fah/Nrf2^−/−^ mice model [45]. Furthermore, regulation of gene expression depends not only on transcriptional level, but also on epigenetic manipulation, such as DNA methylation and histone modifications [46]. Thus, whether NRF2 activation results in increased GSTZ1 expression or not needs further investigation. Our results indicate the importance of GSTZ1 on HCC proliferation through the KEAP1/NRF2 pathway. Accordingly, further studies are required to further elucidate the mechanisms underlying the inhibitory effect of GSTZ1 on HCC tumorigenesis and progression.

## Conclusions

In summary, this study shows that GSTZ1 is downregulated in HCC and may serve as a prognostic marker. Our findings indicate that GSTZ1 deficiency induces oxidative stress, thus activating the KEAP1/NRF2 signaling pathway, which promotes HCC progression. This study shows the effect of GSTZ1 on HCC proliferation and the underlying mechanism, suggesting that modulation of oxidative stress and the NRF2 pathway may be a potential therapeutic strategy for this subset of HCC; however, further studies are required.

## Supplementary information

**Additional file 1: Table S1.** Quantitative RT-PCR Primer Sequences.

**Additional file 2: Figure S1.** pGL3-ARE plasmid map.

## Data Availability

All data generated during this study are included in this article.

## Abbreviations

4-HNE: 4-hydroxy-2-nonenal
ARE: Antioxidant response element
DEN: Diethylnitrosamine
ECAR: Extracellular acidification rate
FAA: Fumarylacetoacetate
GSH: Glutathione
GSTZ1: Glutathione S-transferase zeta 1
HCC: Hepatocellular carcinoma
KEAP1: Kelch-like ECH-associated protein 1
LIHC: Liver hepatocellular carcinoma
MAA: Maleylacetoacetate
NAC: N-acetylcysteine
NRF2: Nuclear erythroid factor 2-related factor 2
OCR: Oxygen consumption rate
TCGA: The Cancer Genome Atlas
