# Supplementary material for: GSTZ1 deficiency promotes hepatocellular carcinoma proliferation via activation of the KEAP1/NRF2 pathway
Source: J Exp Clin Cancer Res. 2019 Oct 30;38:438. doi: 10.1186/s13046-019-1459-6 (PMC6822483; doi:10.1186/s13046-019-1459-6)
Supplement: Supplementary file 1 — Additional file 1: Table S1. Quantitative RT-PCR Primer Sequences. [file 13046_2019_1459_MOESM1_ESM.docx]

**Table S1 Quantitative RT-PCR Primer Sequences**

| Name | Accession Number | Source | Sequence (5’-3’) |
| --- | --- | --- | --- |
| *Gstz1*  (Human) | NM_145870 | TsingKe Biological Technology | Forward: CCTGAAGCAAGTGGGAGAGG  Reverse: TGATGGTAGGGTAGGGGGTG |
| *Nqo1*  (Human) | NM_000903 | TsingKe Biological Technology | Forward: CCAGCAGACGCCCGAAT  Reverse: CCAAGTGATGGCCCACAGA |
| *Ho-1*  (Human) | NM_002133 | TsingKe Biological Technology | Forward: AACTTTCAGAAGGGCCAGGT  Reverse: CTGGGCTCTCCTTGTTGC |
| *G6pd*  (Human) | NM_000402 | TsingKe Biological Technology | Forward: TGACCTGGCCAAGAAGAAGA  Reverse: CAAAGAAGTCCTCCAGCTTG |
| *Gclm*  (Human) | NM_002061 | TsingKe Biological Technology | Forward: GACAAAACACAGTTGGAACAGC  Reverse: CAGTCAAATCTGGTGGCATC |
| *Gstp1*  (Human) | NM_000852 | TsingKe Biological Technology | Forward: CCCTACACCGTGGTCTATTTCC  Reverse: CAGGAGGCTTTGAGTGAGC |
| *Mrp2*  (Human) | NM_000392 | TsingKe Biological Technology | Forward: ACGGGCACATCACCATCAAG  Reverse: CTCCAGGCAGCATTTCCAAG |
| *Txn*  (Human) | NM_003329 | TsingKe Biological Technology | Forward: CCCTTTCTTTCATTCCCTCTCTG  Reverse: ATTCACCCACCTTTTGTCCCT |
| *β-actin*  (Human) | NM_001101 | TsingKe Biological Technology | Forward: AGGCCAACCGCGAGAAGATGACC  Reverse: GAAGTCCAGGGCGACGTAGCAC |
| *Nqo1*  (Mouse) | NM_008706 | TsingKe Biological Technology | Forward: AGCCCAGATATTGTGGCCG  Reverse: CCTTTCAGAATGGCTGGCAC |
| *β-actin*  (Mouse) | NM_007393 | TsingKe Biological Technology | Forward: CGTTCAATACCCCAGCCATG  Reverse: GACCCCGTCACCAGAGTCC |
