# Supplementary figures and images for: GSTZ1 deficiency promotes hepatocellular carcinoma proliferation via activation of the KEAP1/NRF2 pathway
Source: J Exp Clin Cancer Res. 2019 Oct 30;38:438. doi: 10.1186/s13046-019-1459-6 (PMC6822483; doi:10.1186/s13046-019-1459-6)

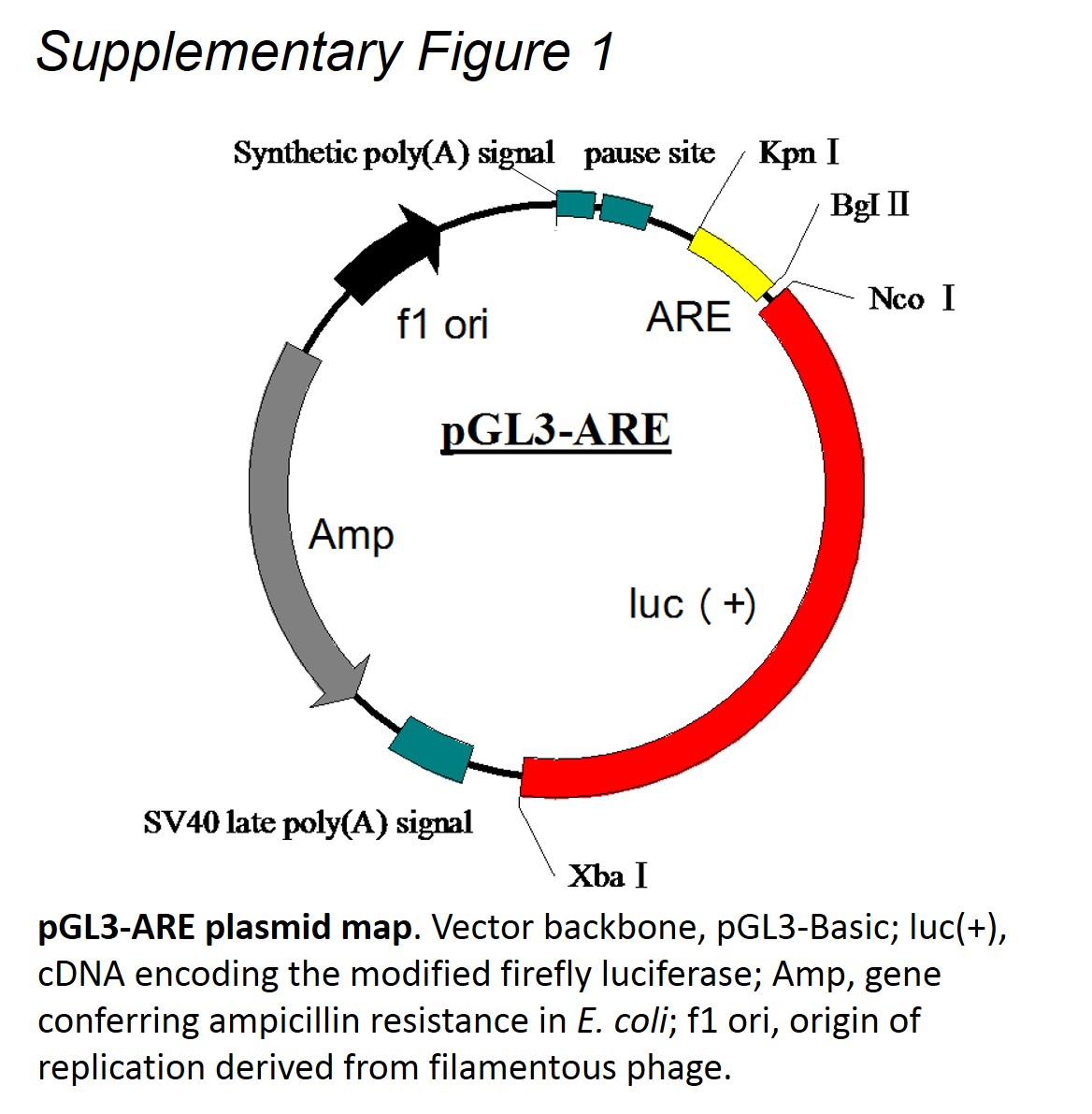

Supplement: Supplementary file 2 — Additional file 2: Figure S1. pGL3-ARE plasmid map. [file 13046_2019_1459_MOESM2_ESM.jpg]
